# An efficient ptychography reconstruction strategy through fine-tuning of large pre-trained deep learning model

**DOI:** 10.1016/j.isci.2023.108420

**Published:** 2023-11-10

**Authors:** Xinyu Pan, Shuo Wang, Zhongzheng Zhou, Liang Zhou, Peng Liu, Chun Li, Wenhui Wang, Chenglong Zhang, Yuhui Dong, Yi Zhang

**Affiliations:** 1 Beijing Synchrotron Radiation Facility, Institute of High Energy Physics, Chinese Academy of Sciences, Beijing 100049, China; 2 University of Chinese Academy of Sciences, Beijing 100049, China; 3 Spallation Neutron Source Science Center, Dongguan, Guangdong 523803, China

**Keywords:** Physics, Optical physics, Computational physics

## Abstract

With pre-trained large models and their associated fine-tuning paradigms being constantly applied in deep learning, the performance of large models achieves a dramatic boost, mostly owing to the improvements on both data quantity and quality. Next-generation synchrotron light sources offer ultra-bright and highly coherent X-rays, which are becoming one of the largest data sources for scientific experiments. As one of the most data-intensive scanning-based imaging methodologies, ptychography produces an immense amount of data, making the adoption of large deep learning models possible. Here, we introduce and refine the architecture of a neural network model to improve the reconstruction performance, through fine-tuning large pre-trained model using a variety of datasets. The pre-trained model exhibits remarkable generalization capability, while the fine-tuning strategy enhances the reconstruction quality. We anticipate this work will contribute to the advancement of deep learning methods in ptychography, as well as in broader coherent diffraction imaging methodologies in future.

## Highlights

• A neural network model design for efficient reconstruction of ptychography data
• Pre-training models and fine-tuning strategies suitable for ptychography are presented
• Network can improve ptychography data reconstruction by reducing overlap rate

## Graphical abstract

**Figure:**
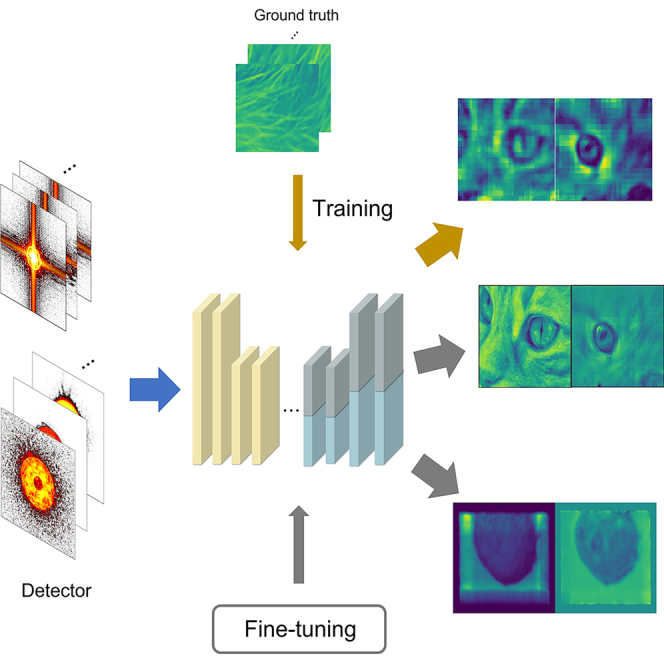

## Introduction

X-ray ptychography, a synchrotron coherent imaging technique that is theoretically capable of achieving diffraction-limited resolution, has been widely used in materials,^1^^,^^2^^,^^3^ life sciences,^4^^,^^5^ and other scientific fields.^6^^,^^7^^,^^8^ Benefiting from the high brightness and excellent coherence nature of next-generation synchrotron sources, ptychography is reaching new levels of application scenarios. For example, the emergence of new ptychography-based imaging technologies, including resonant ptychography,^9^^,^^10^ ptycho-tomography,^11^^,^^12^^,^^13^ and *in situ* ptychography,^14^^,^^15^ allows for multi-dimensional analysis, fine structure study and functional characterization of large-volume samples with improved temporal resolution. Despite the potential of these new ptychography methods, significant challenges remain in the algorithmic and software domain to address their online data analysis requirements.^16^

Phase retrieval and ptychographic sample reconstruction are inherently one of the most difficult tasks in synchrotron radiation methodologies. As a relatively time-consuming scanning imaging technique, one of the main goals of ptychography is to achieve real-time analysis. Although the traditional physical reconstruction process is maturely developed,^17^^,^^18^^,^^19^^,^^20^^,^^21^^,^^22^^,^^23^ it is currently difficult to achieve highly precise reconstruction for new high-throughput multidimensional characterizations. Besides, the reconstruction algorithms are limited by numerous deficiencies, such as weak tolerance to interference, sensitivity to initial guesses, and the tendency to fall into local optima which may prevent obtaining a relatively accurate reconstruction. In recent years, convolutional neural networks (CNNs) have achieved remarkable success in the field of image processing, such as image noise reduction,^24^ image restoration,^25^ image super-resolution,^26^ and object detection,^27^ etc. Due to its efficiency and accuracy, CNN has been gradually adopted in the field of ptychography. Since 2017, CNNs have obtained good results on ptychography reconstruction.^28^^,^^29^ Later, PtychoNet^30^ and PtychoNN^31^ further improved the network architecture and achieved decent results under low overlap rates. In 2022, Welker^32^ and others constructed Deep Iterative Projections (DIP) neural network after analyzing the similarities between the speech signal processing domain and ptychography, which better reconstructed the simulated objects. Recently, deep learning has also made significant progress in ptycho-tomography and dose reduction.^33^^,^^34^

The aforementioned approaches focus on network architecture and methods optimization, aiming to obtain better reconstruction quality and effectiveness. In the realm of deep learning, significant advancements have been made in natural language processing (NLP) and computer vision (CV) through the utilization of data-driven large-scale models. These models leverage vast amounts of high-quality data to enable neural networks to learn the underlying data logics, accelerating the development of pre-trained models. Notably, pre-trained models have demonstrated remarkable performance across diverse downstream tasks, as evidenced by the success of models like ChatGPT and other language-based architectures.^35^^,^^36^ In addition, for different downstream tasks, the fine-tuning approach leads to targeted tuning of the pre-trained model to obtain better performance. For example, the fine-tuning based on LLaMA pre-training model leads to increased performance in cross-language applications involving Chinese.^37^ Looking ahead, the advent of fourth-generation synchrotron light sources would generate enormous volume of data for coherent diffraction imaging,^38^ which provides a unique opportunity to explore the application of large-scale models in the field of coherent diffraction, particularly when tackling challenges related to reconstruction. By leveraging the potential of training big models in this field, we can harness the power of data-driven approaches to enhance the effectiveness of coherent diffraction imaging techniques.

While deep learning algorithms have made significant progress in the field of coherent diffraction imaging, existing neural networks still require continuous optimization and improvement to achieve better performance in ptychography as data volume continue to scale up. In this paper, to enhance the performance of neural networks in ptychography, we first propose a lightweight and efficient network model (PtyNet-S). This model serves as a foundation that can be further improved to larger model (PtyNet-B) to accommodate larger-scale data training, thereby providing more accurate and robust reconstructions. Then, we introduce a novel fine-tuning method based on the pre-trained neural network model. This fine-tuning approach leads to better reconstruction quality and yields favorable results across different overlap rates. By leveraging the advantages of both pre-training and fine-tuning, our method presents a promising approach to the advancement of ptychography.

## Results

### Architectural improvements of small neural network

**Figure 1:**
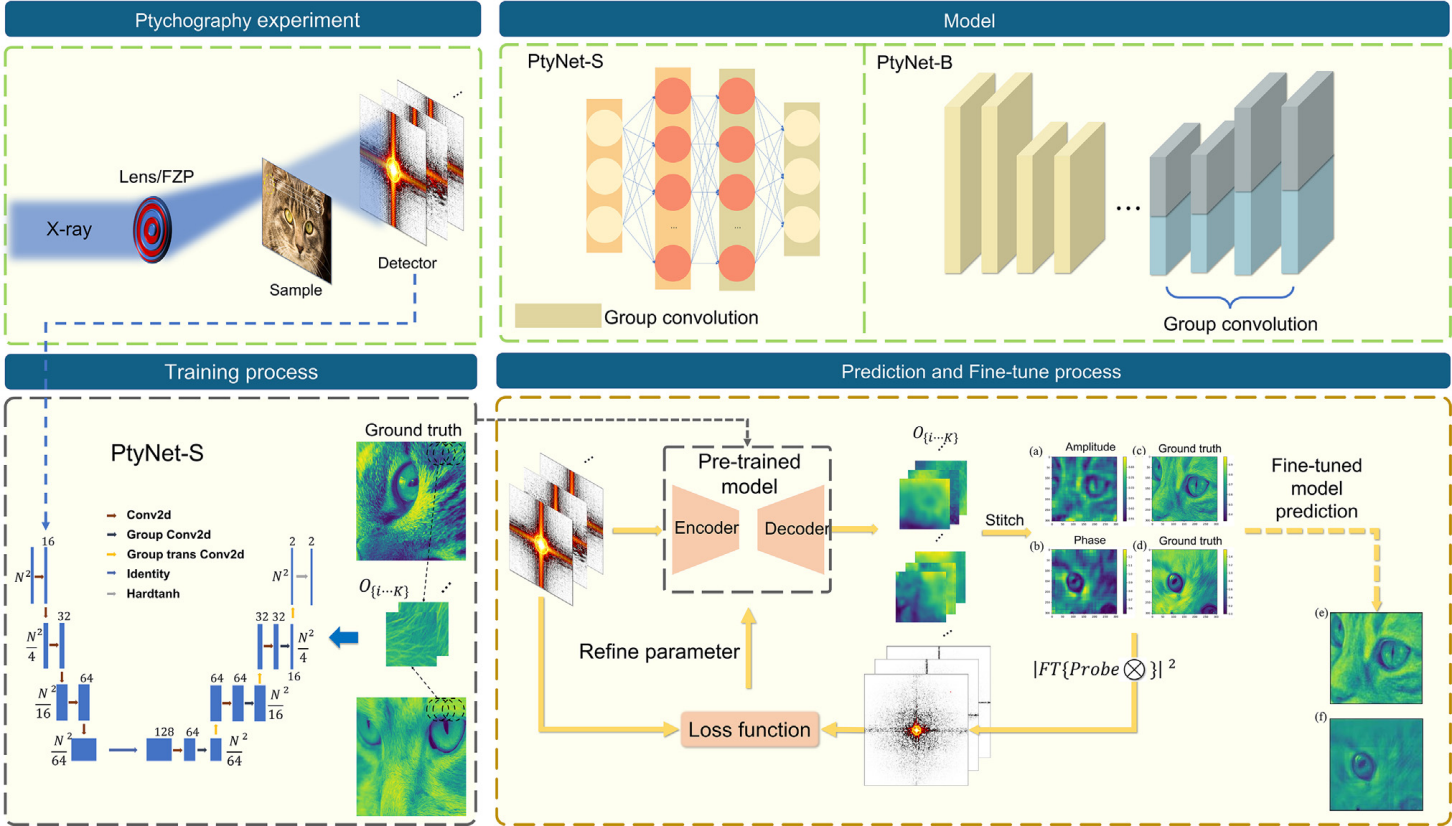
This figure illustrates the essential stages in the PtyNet-S workflow, encompassing training, prediction, and fine-tuning (a and b) in prediction and fine-tune process part depict the outcomes of PtyNet-S in reconstructing both amplitude and phase, while (c) and (d) showcase the distributions of simulated objects. PtyNet-S undergoes supervised training, relying on real object distributions as its foundation. After training, the model can predict corresponding amplitude and phase distributions for the input diffraction pattern at each scanning position. These predictions are subsequently stitched to generate the final distribution prediction. The fine-tuning process involves updating the neural network model using diffractograms obtained from known probes and get better reconstruction results.

By using CNN to recover ptychography images, PtychoNN and PtychoNet have made substantial progress. Motivated by this, we designed a lightweight and effective convolutional network architecture named PtyNet-S. The main structure of the proposed PtyNet-S is shown in Figure 1. In practice, we use group convolution instead of the two-branch decoder structure from PtychoNN. Group convolution allows different convolution kernels to extract information efficiently from different feature maps without affecting each other (see architecture of neural networks in STAR methods for details). This approach avoids the excessive computational resources associated with double branching and also reduces the number of parameters in the model. The input to the model is a single diffraction pattern, and the output is the amplitude and phase of the reconstructed object corresponding to the diffraction pattern. When testing on the open-source dataset, PtyNet-S shows better phase reconstruction performance with only 320,000 parameters comparing to PtychoNN which has 1.2 million parameters (see Table 1 for details).

**Table 1:** Performance comparison of the three models on the same dataset

	Parameters/Thousand	MSE(Phase)	Computational resource ratio (A100)/%	GPU memory/GB
PtychoNN	5512	0.0885	86%	2.7
PtychoNet	1247	0.1633	80%	2.3
PtyNet-S	325	0.0852	46%	2.0

We trained the PtyNet-S network using a small simulated training dataset (see STAR methods for details on training) and then put the cat face data from the test dataset into PtyNet-S for inference prediction after data preprocessing. The obtained experimental results are shown in Figure 1. The neural network learns the mapping relationship $F_{\theta }$ from the diffraction domain to the real data domain, and the real-time online processing of the ptychography experiment can be performed due to the lightweight design of the network.

It can be seen from Figure 1 that the neural network can learn the mapping process from the diffraction to the real object. During the convolution process, the high frequency information of the data are lost and the low frequency information is retained which is thus recovered by the decoder. The artifacts showing pixelated effects are caused by the network’s limitations when predicting final results with absolute accuracy, and by the stitching process (see Figure S7). This is mainly because that the detailed texture of the cat face data are too complex, and for scans of similar regions, the results predicted by the network will behave differently in the distribution of high frequency regions, while with little difference in the distribution of low frequency regions. This will lead to inconsistency in the overlapping regions of the scans predicted by the neural network, and the grid artifacts will appear after the stitching process.

**Figure 2:**
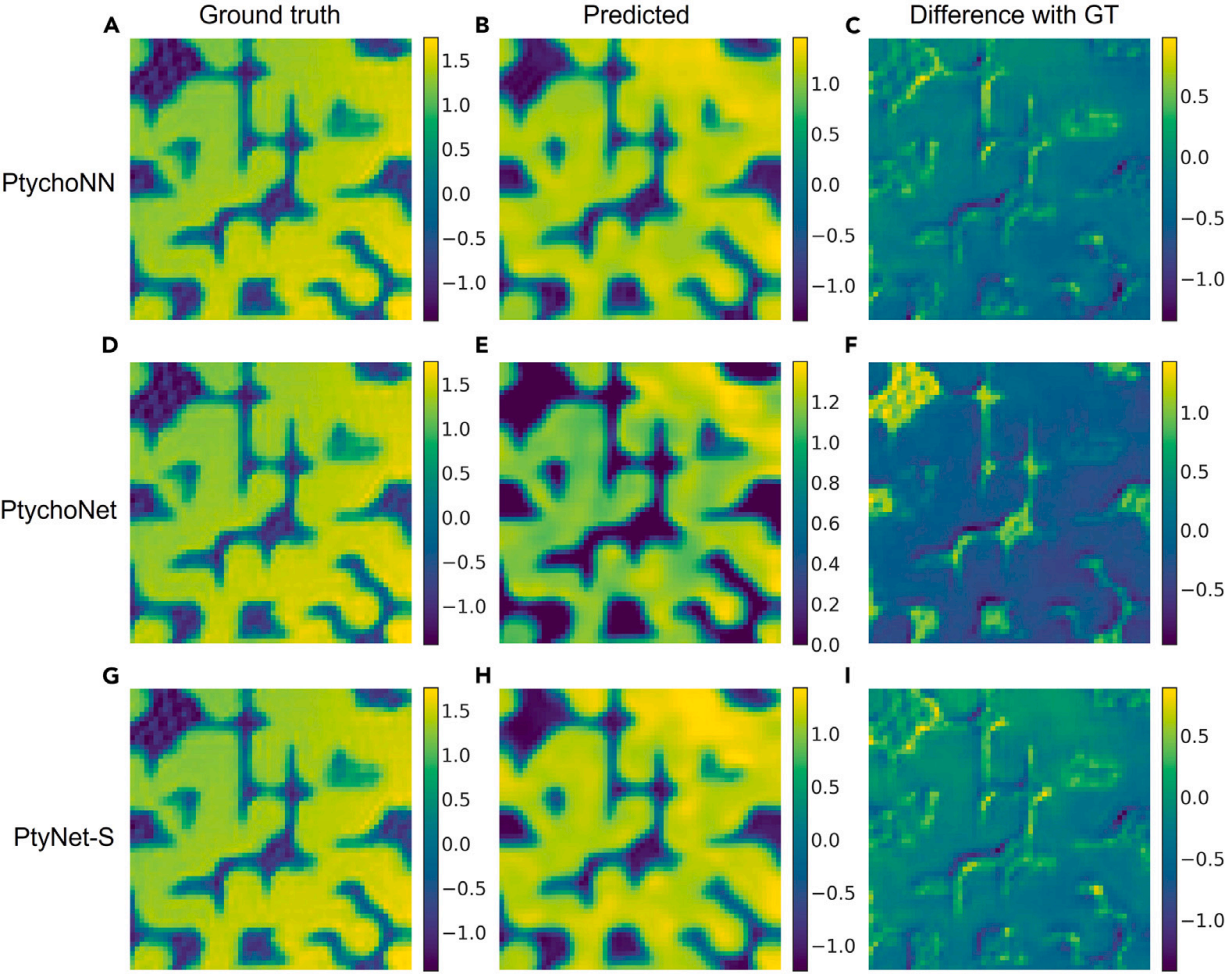
Phase reconstruction results of the three models in tungsten test pattern (A, D, and G) are the ground truth of the tungsten test pattern. (B, E, and H) are the prediction results after PtychoNN, PtychoNet, and PtyNet-S are output and stitched together, respectively. (C, F, and I) are the differences between the prediction results and the ground truth, respectively. PtyNet-S and PtychoNN have similar performance, and both predict better than PtychoNet, while the PtychoNet predicts better on the sample profile.

Benchmark with previous models: to compare the difference between PtyNet-S, PtychoNet, and PtychoNN, we use the same training strategy and testing dataset to measure the effectiveness and accuracy of both models. The public experimental dataset^31^ measured on tungsten sample which has been published in the PtychoNN paper. As shown in Figure 2. Meanwhile, we compared the deviation of the three models on the experimental data, the number of model parameters, the computational resource utilization ratio, and the amount of the graphics memory occupied by the models, are shown in Table 1, which shows that PtyNet-S yields better results. In addition we also compared the ROI of the simulated data reconstruction results, as detailed in the supplemental information.

### Pre-trained models and generalization capabilities

As shown in Figures 1 and 2, the model has good generalization ability even with a small dataset, which has the potential to build a pre-trained model with large dataset in the field of ptychography and even extent to general coherent X-ray diffraction methods. In recent years, the excellent performance of neural networks in the image processing domain highly depends on the growth of scale of the network parameters, as well as the amount and quality of data. For example, the SAM^39^ introduced by Meta AI uses data comprising as much as 1.1 billion images and have 10 billion parameters(base model), showing excellent segmentation results. The results of SAM demonstrate that pre-trained networks by using large dataset have strong representational capacity. The improvement on quality and quantity of the data also requires the neural network be able to extract to the feature of more input data, and an increase of model size is an effective way to improve the performance. Therefore, further improved the architecture of PtyNet-S and build a larger pre-trained PtyNet-B using simulated data to achieve better reconstruction results. To accommodate the large amount of data (about 60000 diffractions), we modified the convolutional, downsampling and upsampling layers of PtyNet-B (Figure S5). Besides, we investigated the reconstruction performance of PtyNet-B by varying the overlap rates (75%, 50%, 25%, 0%) in the production of the simulated data.

**Figure 3:**
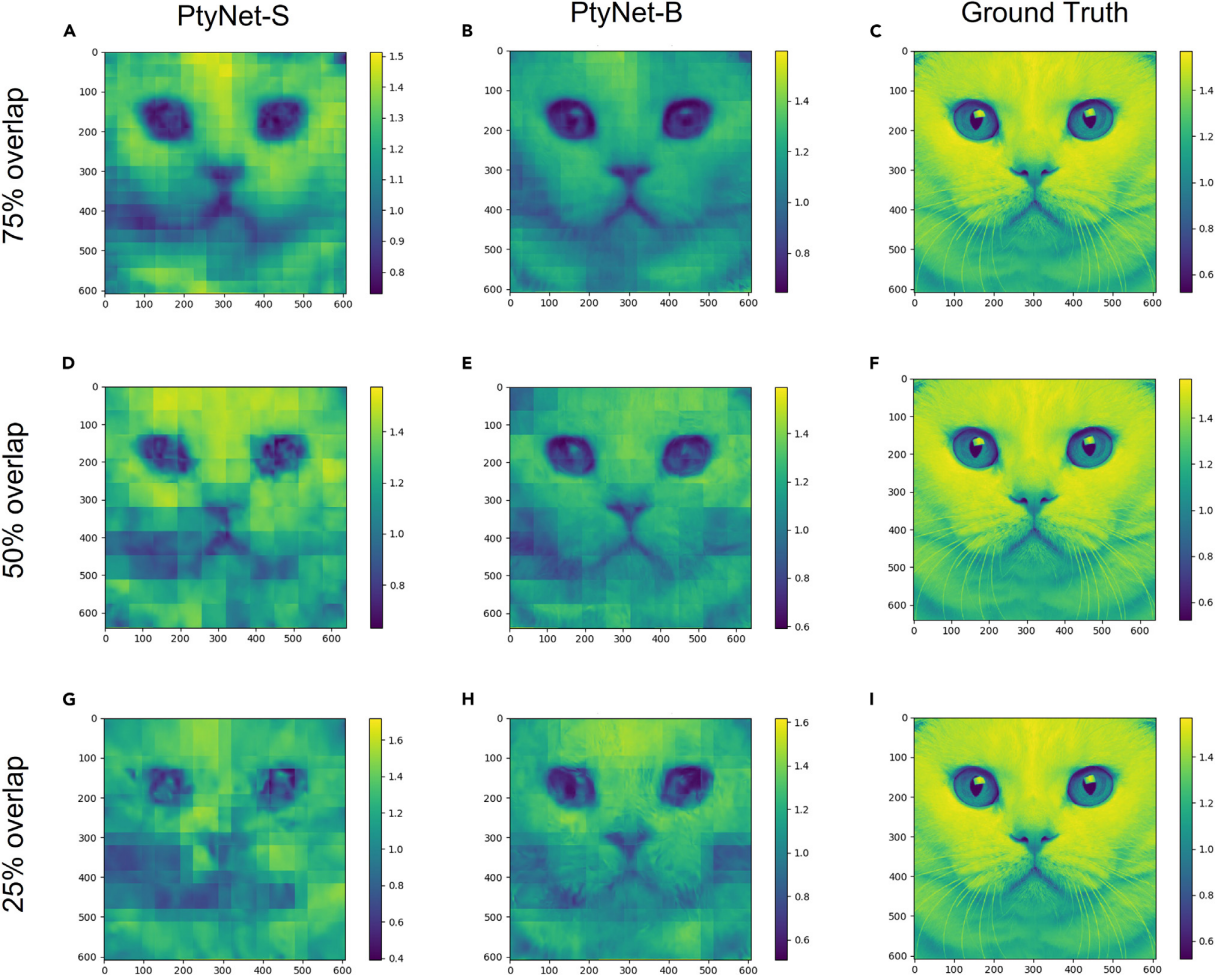
Results of the prediction of the phases in the testing set using the PtyNet-S and PtyNet-B with different overlap rates (A–C), (D–F), and (G–I) are the results of stitched results after PtyNet-S prediction, the results of stitched results after PtyNet-B prediction, and ground truth, respectively.

With the improved network architecture, the PtyNet-B has more parameters and can learn more of the intrinsic logic of the data. As shown in Figure 3 and Table 2, the performance of the PtyNet-B is improved compared to the previous PtyNet-S in all overlap rates. However, during the convolution process, there are still some extents of loss in high frequency information even use PtyNet-B, while the low frequency information is retained and can be recovered subsequently by the decoder (see Figure S8 for visualization).

**Table 2:** Performance comparison of PtyNet-S and PtyNet-B

Model	% Overlap								
	75			50			25		
	PSNR	SSIM	MSE	PSNR	SSIM	MSE	PSNR	SSIM	MSE
PtyNet-S	**12.919**	0.793	0.010	**14.349**	**0.816**	**0.011**	12.353	0.791	0.041
PtyNet-B	12.419	**0.802**	**0.009**	12.434	0.799	0.021	**12.933**	**0.797**	**0.025**

The bold values in PSNR, SSIM, and MSE indicate better results.

### Fine-tuning results

In the field of deep learning, specific downstream tasks such as medical image segmentation can be tackled by leveraging fine-tuning techniques that pre-train the model. The pre-training allows the model to acquire generalized knowledge, which can then be adapted to boost performance on the target task during fine-tuning. Through this transfer learning approach, the model’s capabilities on specialized downstream applications can be significantly improved, yielding higher accuracy compared to training from scratch.

**Figure 4:**
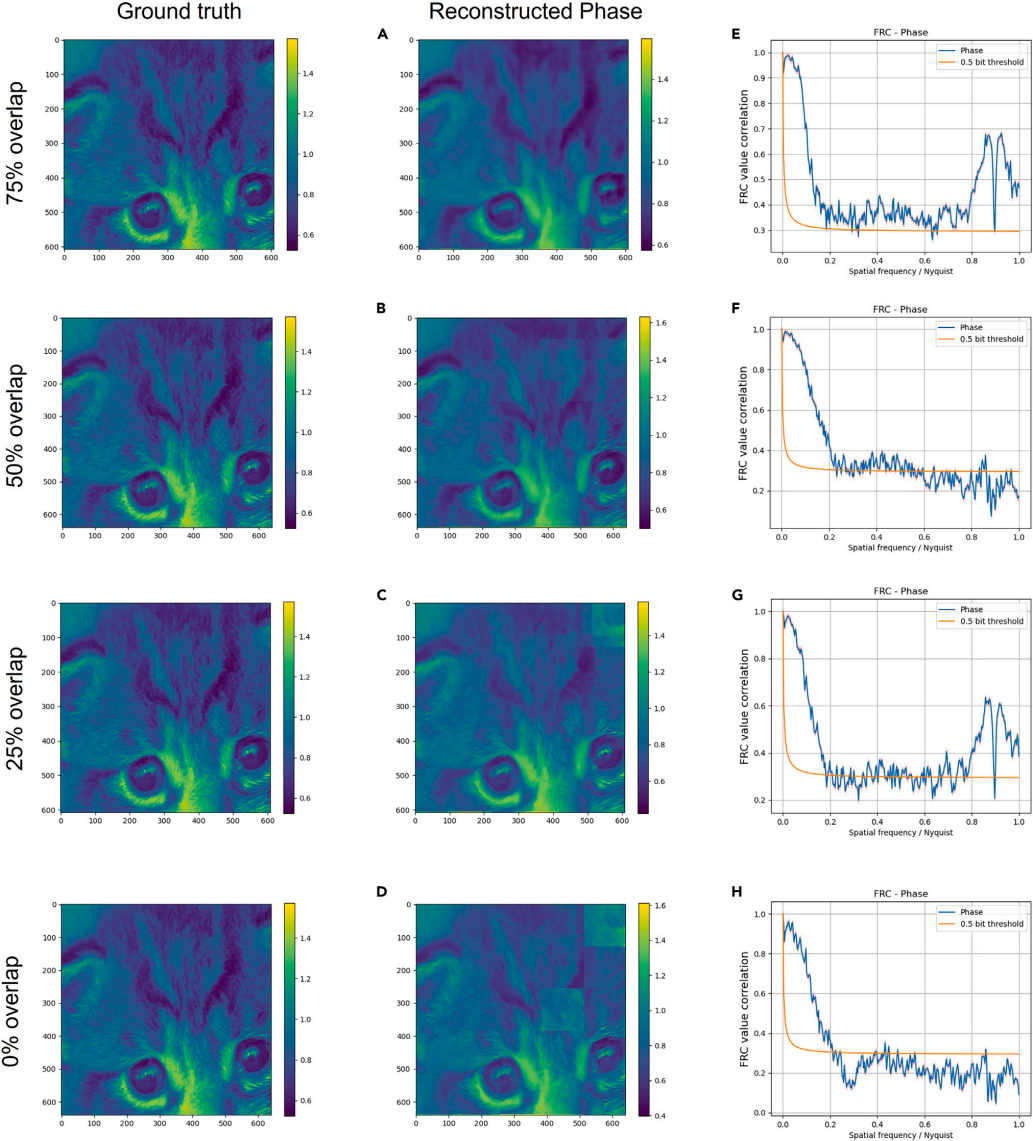
Results of fine-tuning phase for different overlap rates (A–D) show the results of fine-tuning the neural network with different overlap rates. (E–H) show the corresponding Fourier ring correlation (FRC) curves.

We use the pre-trained PtyNet-B as the initialization for fine-tuning. The image quality is optimized by fine-tuning for different objects (see STAR methods for details of the fine-tuning process). We randomly select a cat face image in the testing dataset as the amplitude and phase (the reason for not using two randomly selected images is that the distribution of amplitude and phase of the samples is consistent in the experiment), and the results obtained by fine-tuning the scanned data with different overlap rates are shown in Figure 4.

As illustrated in Figure 4, the fine-tuning method can improve better prediction of the neural network for different samples and it also has good prediction results at low overlap rates. In the Fourier ring correlation (FRC) curves in (e)–(g) of Figure 4, there are peaks in the high frequency region. We believe this is due to various factors affecting the accuracy of the neural network reconstruction, such as those caused by the stitching method. As a result, the final object obtained presents tile-shaped artifacts locally, which may cause the peak in the FRC curves. In addition, at the highest overlap rate of 75%, the input diffraction pattern is 256 × 128× 128, and it took about less than 90 s to fine-tune all the parameters of the neural network (with a single Nvidia A100 GPU). In order to verify the capability of the fine-tuning method on different types of data, we used both simulated data (human face) and experimental data^40^ (fluid catalytic cracking catalyst particle, FCC) for testing. Using a 50% scanning overlap rate and regular raster scan method, a total number of 81 diffraction patterns (256 × 256 pixels) are acquired from the simulated human face. Using a 51% scanning overlap rate and Fermat spiral trajectory scan method, a total number of 2347 diffraction patterns (512× 512 pixels) are acquired in the experiment.

**Figure 5:**
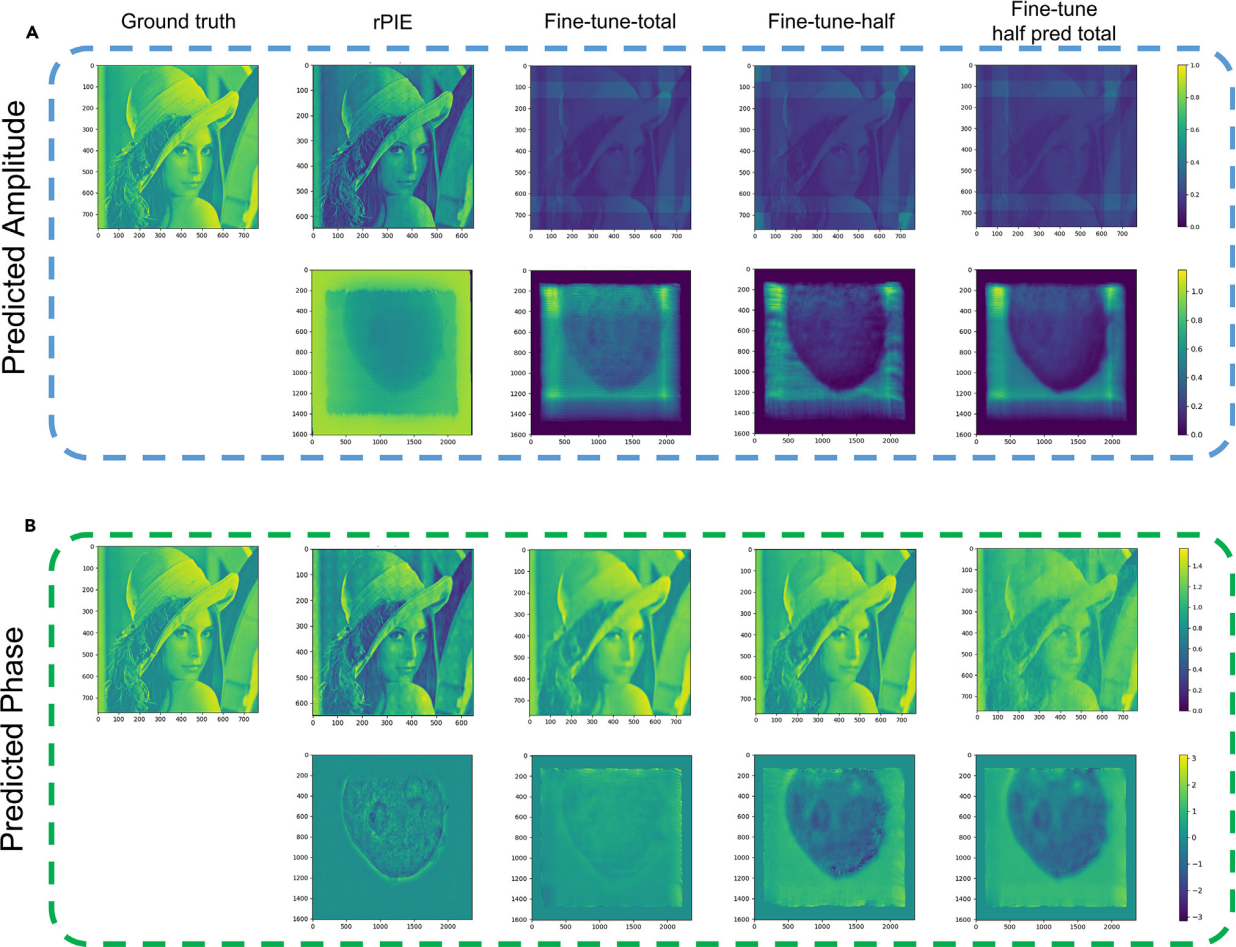
Results of 400 epochs of fine-tuning using neural networks for different data types (A) Shows the amplitude results predicted by the model after fine-tuning. (B) Shows the phase results predicted by the model after fine-tuning. Fine-tune-total indicates that all the data were fine-tuned. Fine-tune-half indicates that half of the data were fine-tuned to further reduce the overlap. Fine-tune half pred total indicates that the model with half of the data fine-tuned was used to predict the entire dataset.

It is worth noting that when using the FCC particle data for fine-tuning, we only used the initial guess probes in the fine-tuning process because the real probes are not given in the public dataset (only the initial guess probes) and the probes reconstructed by rPIE are not ideal. The results in Figure 5 show that the fine-tuning method can still obtain good reconstruction results for datasets of different types and sizes. We noted that the results reconstructed after fine-tuning all the data are worse than those reconstructed after fine-tuning half the data. We believe this is because for the same number of fine-tuning epochs, reducing the amount of data by half allows the neural network to fit to the features of the samples faster thus allowing for a reduction in the overlap rate limitation. Also less data allow the network to learn higher frequency information in more epochs to recover the details of the sample. The training using large amount of data can give the model good generalization ability and initialization, while the fine-tuning method allows the model to learn in detail for different data types, and improves the generalization ability even further.

## Discussion

### Noise interference

**Figure 6:**
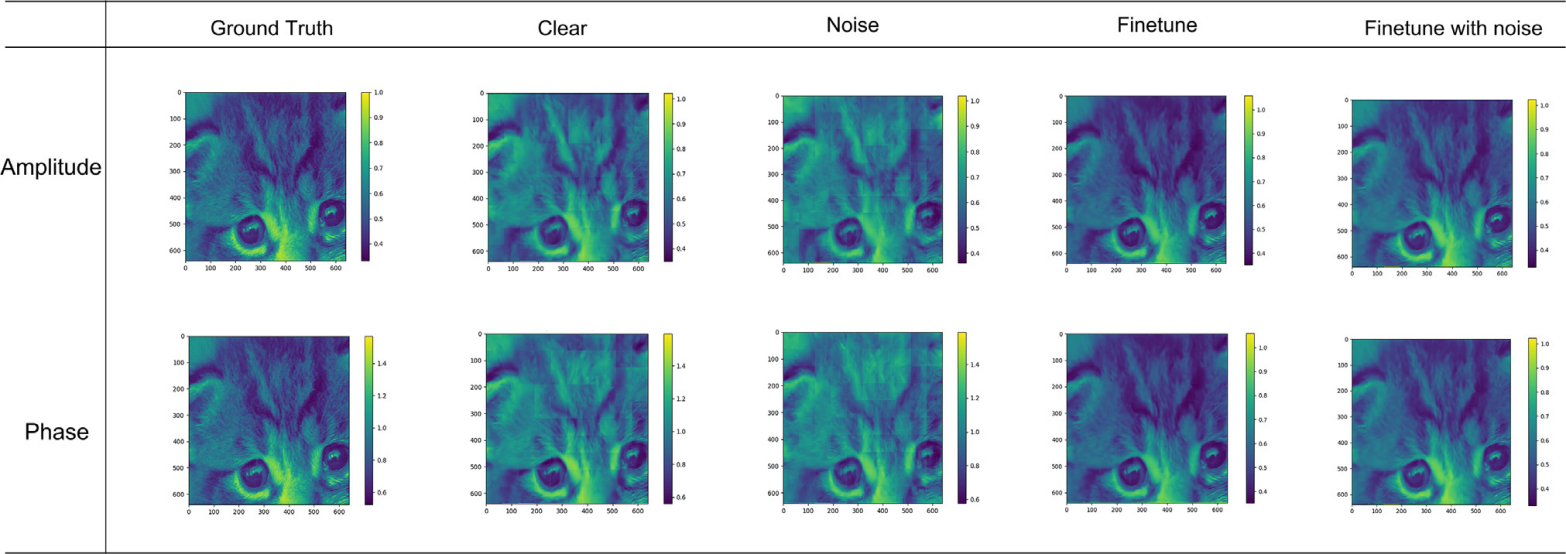
Reconstruction results of PtyNet-B in noiseless and noisy conditions.

In ptychography experiments, due to the small focusing probe size and large photon counts on the detector, the Gaussian noise in the detector background does not have a significant impact on the signal, while the effect of Poisson noise cannot be ignored. Since the self-encoder of the CNN has a natural tolerance to high frequency noise, we added Poisson noise to the data. We also considered the probe with a focus shift of about 5 μm in the object plane to better emulate real experimental scenarios. The results are shown in Figure 6.

The test results show that the neural network has strong robustness under the influence of noise. However, the fine-tuning results in Table 3 with noise are better than the results without noise. This is because the neural network mainly recovers low and medium frequency information, and the high frequency information is largely lost under the effect of convolution after mixing with noise. And in the result with noise, the high frequency information corresponds to most of the distortion.

**Table 3:** Noise influence without and with fine-tuning on model performance

Poisson Noise	Ground Truth			Clear			Noise			Fine tune with clear			Fine tune with noise		
	PSNR	SSIM	MSE	PSNR	SSIM	MSE	PSNR	SSIM	MSE	PSNR	SSIM	MSE	PSNR	SSIM	MSE
Amplitude	∖	1	0	**19.603**	**0.714**	**0.005**	17.710	0.689	0.009	22.714	0.753	0.002	**25.312**	**0.756**	**0.001**
Phase	∖	1	0	**17.694**	**0.691**	**0.009**	16.384	0.670	0.013	22.713	0.748	0.002	**25.314**	**0.764**	**0.001**

The bold values in PSNR, SSIM, and MSE indicate better results.

The noise can destroy the high frequency signal and make traditional algorithms fail to reconstruct the high-resolution components. Neural networks can reconstruct the low and medium frequency information better, while the deficiency in high frequency information reconstruction is still not resolved sufficiently. In the future, the solution of noise is one of the key factors to obtain high quality reconstruction in ptychography. The influence of noise in neural networks and how optimization can be done in noise presence is a focus of our future endeavor.

### Probe issues

**Figure 7:**
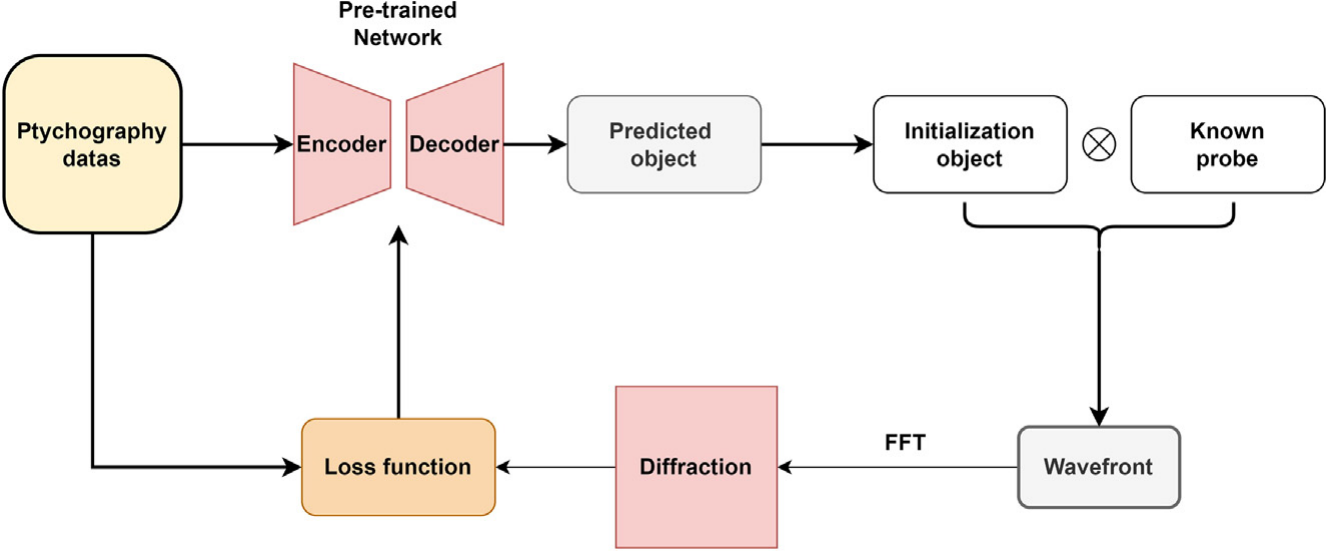
Schematic diagram of the fine-tuning workflow The predicted object is obtained from the ptychography data after pre-trained network prediction. Then, the wavefront is obtained by interaction with the probe, and then the diffraction pattern is obtained by forward propagation. The fine-tuned network can make the predicted diffraction pattern as close as possible to the real diffraction pattern.

In results section, fine-tuning improves the quality of the final neural network reconstruction. However, during the fine-tuning process (shown in the Figure 7), the neural network requires known probes as input, which is only available in simulation. In real experimental situations, the probes are usually not readily available. Therefore, the fine-tuning technique needs to obtain the distribution of probes in advance. Unfortunately, the size and shape of the probe may change during the experiment due to instrumentation instabilities, position errors, and other unpredictable factors. The fine-tuning strategy can be applied if it is possible to use a standard sample to measure the probe in advance of the experiment, and it is assumed that the probe will not change after changing the sample to be measured. In the future, we will explore how to add adaptively tunable probes to the neural network and how to update the probes based on the results of the model.

**Figure 8:**
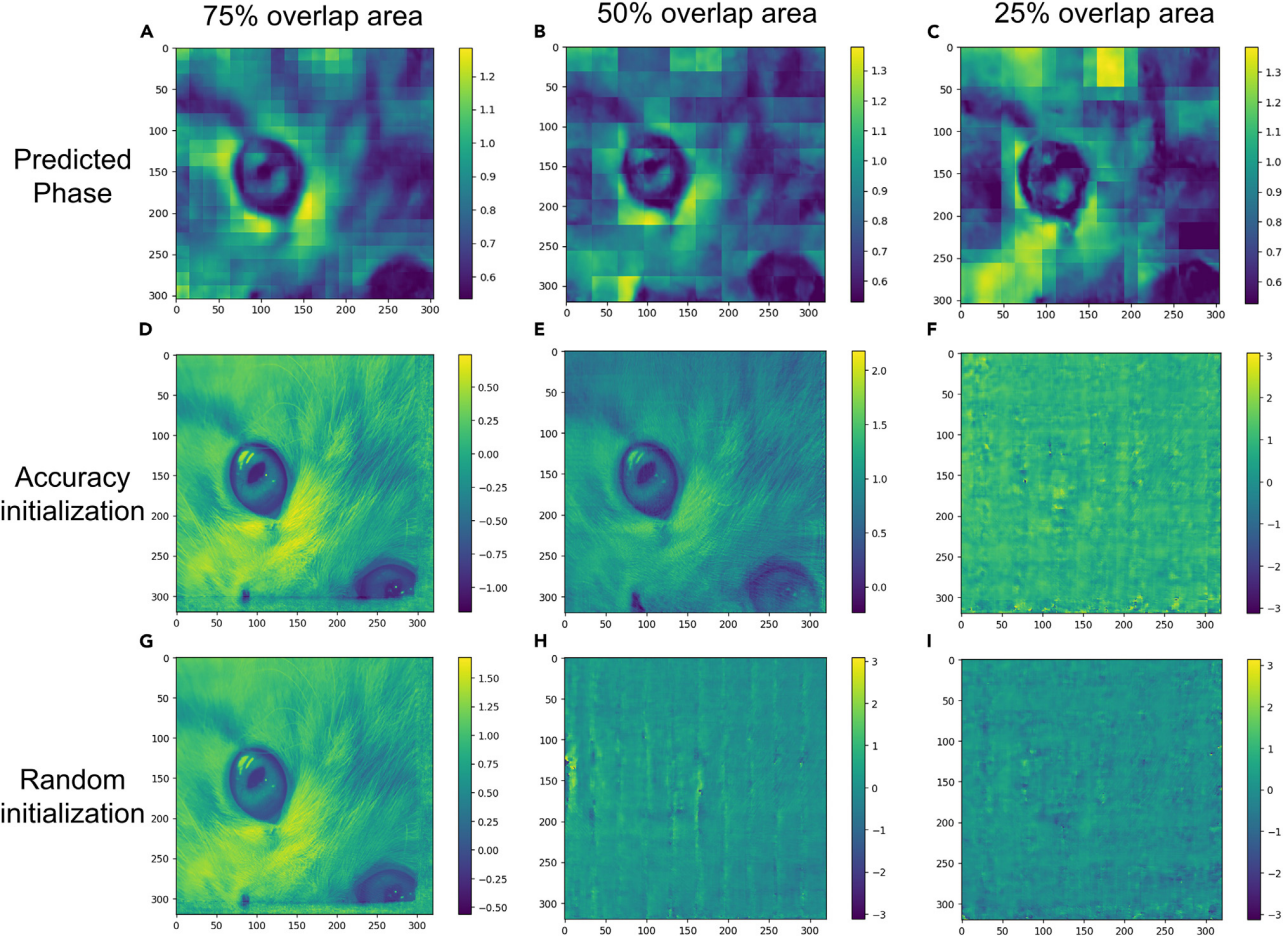
The output of the neural network is used as an initial guess for the iterative algorithm (A–C) show the phase values of the network predictions at different overlap rates as initial guesses for rPIE and ePIE. (D–F) are the results of reconstructing 50 rounds of phases from the network predictions as initial guesses at different overlap rates. (G–I) are the results of reconstructing 50 rounds of phases with random initial guesses at different overlap rates.

**Figure 9:**
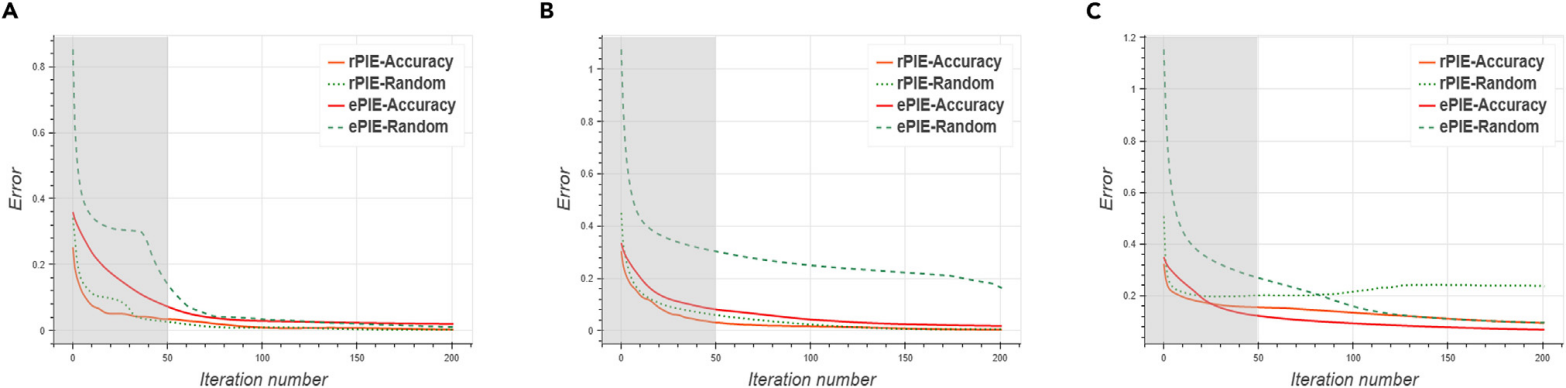
Error curves for the ePIE and rPIE algorithms under accurate initial guessing and random initial guessing (A–C) shows the error curves of the reconstructed objects using different algorithms. From the error curves, it can be seen that the accuracy variant of the initial guesses converges faster.

### High-dimensional ptychography expansion

All the experiments and methods discussed previously are for the reconstruction of two-dimensional objects. However, the thickness of samples for ptychography often cannot be ignored. To obtain a higher resolution, multi-slice reconstruction is required. Although some multi-slice reconstruction methods are already available,^41^^,^^42^^,^^43^ there still remain concerns. For example, there are no definitive criteria on the number of slices and the thickness of the slices needed for multi-slice model reconstruction. The existing ptycho-tomography and resonant ptychography techniques face problems such as low-resolution projection reconstruction and expensive computational cost. Although our deep learning method has good extensibility, the extension to high-dimensional ptychography is another research direction.

### Conclusions

We proposed two neural network architectures: PtyNet-S and PtyNet-B. PtyNet-S reduces memory usage and calculations through optimized design with better performance. It demonstrates good in reconstructing phase information. Building on PtyNet-S, PtyNet-B further expands the model scale and enhances the architecture. By increasing training data five times, PtyNet-B significantly improves final reconstruction quality across varying overlap rates. Finally, we use a fine-tuning approach to further improve the reconstruction quality, achieving satisfactory resolution. We also discuss the influence of noise on the neural network and the effect of probes in the fine-tuning methods. In the proposed reconstruction method, no prior knowledge of overlap is added. Compared with traditional algorithms, deep learning methods can reconstruct relatively better results even at low overlap rates. If it is used as a prior process to support the initial guessing of traditional algorithms, even higher efficiency and accuracy can be obtained (As the results in Figures 8 and 9 show, see STAR Methods for details). We believe a better applicable pre-training model can be obtained by supervised learning method using high quality reconstructed experiment data. The fine-tuning strategy is then applied to further increase the reconstruction resolution of data collected at various experimental scenarios (varied sample and probes). In the future, the fast prediction of neural networks can greatly improve the experimental efficiency in the data processing of ptychography experiments where a huge amount of data are present. In combination with existing software packages such as PyNX,^44^ Ptypy,^45^ etc., it is also applicable to accelerate the computation process for these traditional algorithms to achieve real-time online analysis on ptychography experimental results.

### Limitation of the study

In this study, we propose a strategy for pre-training and fine-tuning ptychography data, aiming to enhance the efficiency and quality of ptychography reconstruction. We introduce an improved neural network model, PtyNet-S, which demonstrates the potential to achieve these improvements. Moreover, by expanding the model parameters, PtyNet-B can serve as a reliable pre-training model, offering a robust initialization for the subsequent fine-tuning process. The pre-training model also exhibits some degree of generalization ability. The limitation of the current study mainly lies in the fact that relying on single probe and distribution of similar pictures for training data restrict network generalization ability. For instance, the pre-training process is limited to cat faces as the training dataset, and the probes are constrained to a specific parameter setting. This may result in a weaker ability of the pre-trained model to generalize and learn mappings, which can be addressed by incorporating greater data diversity. Additionally, our fine-tuning strategy necessitates the use of known probes, which can lead to potentially significant different of diffraction data between the fine-tuned and pre-trained probes. This disparity in data may result in a resolution degradation (e.g., FCC data). However, we recommend customizing the training model to the specific beamline station and incorporating data from different probes during training. Furthermore, automating the loading of different probes for fine-tuning can help alleviate the aforementioned challenges.

## STAR★Methods

### Key resources table

**Table:** 

REAGENT or RESOURCE	SOURCE	IDENTIFIER
**Deposited Data**		

Tungsten test pattern	Argonne National Laboratory	https://github.com/mcherukara/PtychoNN
FCC	Paul Scherrer Institute	https://doi.org/10.6084/m9.figshare.7993247

**Software and algorithms**		

python 3.9.12	Python	https://www.python.org/
pytorch 1.12	Pytorch	https://pytorch.org/
Numpy 1.23.0	Numpy	https://numpy.org/

**Other**		

Source code	Github	https://github.com/paidaxinbao/PtyNet

### Resource availability

#### Lead contact

Further information and requests for resources and reagents should be directed to and will be fulfilled by the lead contact, Yi Zhang(zhangyi88@ihep.ac.cn).

#### Materials availability

This study did not generate new unique reagents.

#### Data and code availability

• The Python code used for network reconstruction in this paper is available at: https://github.com/paidaxinbao/PtyNet.
• The publicly available data can be found at https://zenodo.org/records/10068181. The link contains the trained and fine-tuned model.
• Any additional information required to reanalyze the data reported in this paper is available from the lead contact upon request.

### Experimental model and study participant details

Our study does not use experimental models

### Method details

#### Reconstruction principle of ptychography by neural network

The reconstruction of the sample at each scanning position of Ptychography can be described as an inverse problem by Fourier transform.

In the sample plane, the coherent light interacts with the sample and the wavefront is:

$$\psi_{i}\left(r\right)=P\left(r\right)*O_{i}\left(r\right)$$

Under the far-field approximation, the wavefront in the detector plane can be described as the Fourier transform of the sample plane as follows:

$$\psi_{i}\left(k\right)=F\left[P\left(r\right)*O_{i}\left(r\right)\right]=A_{i}\left(k\right)e^{i\varphi \left(k\right)}$$

where r denotes the real domain and k represents the frequency domain.

The reconstruction process of the sample can be described as:

$$O_{i}\left(r\right)={\frac{1}{P\left(r\right)}F}^{-1}\left[A_{i}\left(k\right)e^{i\varphi \left(k\right)}\right]=F^{-1}\left[\frac{A_{i}\left(k\right)}{P\left(r\right)}e^{i\varphi \left(k\right)}\right]$$

However, due to the missing phase of the detector, the acquired signal is only ${\left|A_{i}\left(k\right)\right|}^{2}$. We expect that the neural network can learn a transformation process that allows the corresponding sample distribution to be reconstructed at each scanning position:

$${\hat{O}}_{i}\left(r\right)=F\left(A_{i}\left(k\right);\theta \right)$$

where θ is the parameter to be learned by the network. The parameters of the neural network are updated by back propagation:

$$\theta_{New}=\theta -\eta \times \frac{\partial L}{\partial \theta }$$

$$\theta \leftarrow \frac{\partial L}{\partial \theta }=\frac{\partial L}{\partial {\hat{O}}_{i}\left(r\right)}\times \frac{\partial {\hat{O}}_{i}\left(r\right)}{\partial \theta }$$

where L is the loss function and η is the learning rate. While updating the parameters of the neural network, the output will be close to the target so that the neural network learns the mapping relationship.

#### Data simulation

For the data used in the reconstruction training, we generated them by simulating real physical processes. We followed coherence experiments conducted at synchrotron light sources and simulated highly focused small spots for the experiments. A highly focused spot has a more complex structure, which implies a higher frequency component in the frequency domain and helps to reconstruct the object image with higher resolution. We used the spot out of focus at a certain position, thus reducing the number of scans. The probe is simulated by Fresnel propagation of a 100 nm-focused and by intercepting a 1 μm size probe at 5 mm out of focus. The wavelength used for the spot simulation was 0.1 nm (see Figure S9 for visualization). To generate images with more similar structures, we cropped the pictures of cats and used only the part containing the cat’s face to generate data. The size of the diffraction pattern by generating is 128 × 128. We used raster scanning to generate diffraction pattern data with overlaps of 75%, 50%, 25%, and 0%, and added about 1% positional error to the scanning process, with scanning steps of 250 nm (32 pixel), 500 nm (64 pixel), 750 nm (96 pixel), and 1 μm (128 pixel), respectively.

The sample simulated consists of two cat faces, one as the amplitude and one as the phase. The transmittance function of the object is expressed as the following equation:

$$O_{n}\left(r\right)=f_{i}\left(r\right)e^{if_{j}\left(r\right)}$$

Considering the absorption of X-rays by the real object, we set the amplitude range of the object to [0, 1]. The phase range of the object is set to [0, $\frac{\pi }{2}$]. For the diffraction generation, we generate the diffraction intensity at the detector plane by Fraunhofer diffraction and use the raster scanning.

$$I={\left|F\left[P\left(r\right)O\left(r-R_{n}\right)\right]\right|}^{2}$$

Subsequently, the target reconstructed by traditional algorithms in this paper is carried out using ePIE^19^ and rPIE,^21^ respectively.

#### Architecture of neural networks

The structure of the PtyNet-S is similar to the U-Net^46^ with a 3-layer encoder block used in the encoder module. The encoder block consists of two 3 × 3 convolutional layer (the second convolution has a step size of 2), followed by a LeakyReLU activation function after each convolutional layer. The decoder part uses a 3-layer decoder block, where each block is composed of two 3 × 3 convolutional kernels and one deconvolutional layer with 4 × 4 kernel size and step size of 2. The convolution operation in the decoder part is performed using group convolution,^47^ where the input tensor is divided into two groups according to the batch, and each group is convolved separately without overlapping, which is more consistent with the physical process of recovering amplitude and phase separately through the neural network (see Figure S5). The LeakyReLU activation function is used after the convolutional layer of the decoder. PtyNet-B consists of 4 coding and 4 decoding layers and uses residual connections for effect enhancement.The specific architecture of PtyNet-B is detailed in Figure S6. The PtyNet-S and PtyNet-B does not use skip-connections, which are commonly used to enhance data transfer from the decoder to the encoder. As we believe that ptychography aims to recover the amplitude and phase distribution of the sample from the diffraction pattern, and the network should learn a mapping relationship rather than relying on skip-connections. Following the network outputs, we will perform a zero assignment operation on the predicted objects to satisfy the oversampling condition.

#### Network training

We choose 110 images of cat faces to generate diffraction patterns as our dataset, 100 of which are used as the training dataset and 10 as the testing dataset. Cat faces have clear contours and hair features existing as texture details and high frequency information, which are suitable for training and testing the network performance. The simulated physical probe is used for Fresnel diffraction to obtain 12800 diffraction patterns of the corresponding scanning positions of each cat face as the input of PtyNet-S. We set the batch size to 16, applied the Adam optimizer,^48^ and used a cyclic learning rate (starting learning rate of 2e-4).^49^ We used the MSE as the loss function for back propagation to update the parameter of network. 1000 epochs were performed on an Nvidia A100 (80G), and it took approximately 3.2 h.

For the dataset of PtyNet-B, we use the same method as above to produce the dataset. We chose different cat face images for which a ptychography simulation process with 75%, 50%, 25%, and 0% overlap was randomly performed to generate diffraction patterns. Then, we balance the data according to different overlap rates, and added a limit on the number of probes randomly jittering, as well as on the number of photons in order to more closely match the experimental scenarios. We set the batch size to 64, applied the Adam optimizer, and used a cyclic learning rate. 1000 epochs were performed on an Nvidia A100 (80G), taking approximately 23.4 h.

#### Fine-tuning method

In this work, we propose a fine-tuning strategy that allows the neural network to reconstruct different objects in high quality. The workflow of fine-tuning is shown in Figure 7. First, we input the data which need to be fine-tuned into the neural network. Then, the exit wavefront is obtained by interacting with the probe and predicted object. Probes are required to be known. The exit wavefront is forward propagated to obtain the diffraction signal in the diffraction plane. The predicted diffraction signal is input to the loss function:

$${\hat{\psi }}_{i}=P_{known}\times F_{\theta }\left(\sqrt{I_{real}}\right)$$

$$L=\frac{1}{N^{2}}\left|{\left|FFT\left({\hat{\psi }}_{i}\right)\right|}^{2}-I_{real}\right|$$

with the input real diffraction signal updating some parameters of the neural network. The reason for choosing L1 loss as the loss function is that it is the same as the R factor. For the fine-tuning part, we use the Adam optimizer with a stepped learning rate (learning rate decays by half every 20% epoch, starting with a learning rate of 1e-4). The number of rounds of fine-tuning can be adjusted. In this article, for the simulated data, fine-tuning 300 epochs can get good reconstruction results and for the experimental data we fine-tuned 400 to 500 epochs.

#### Integration with traditional algorithms

In traditional algorithms, different scanning overlap rates can have a critical impact on the reconstruction quality of the object.^50^ Generally, more than 50% overlap is required to obtain better results. Also, iterative algorithms rely on the selection of initial guesses when reconstructing objects. A more accurate initial guess can speed up the convergence process. When computational resources are not available for training or fine-tuning larger models, a combination of PtyNet-S and traditional algorithms can be used to improve the reconstruction efficiency and reconstruction quality.

As shown in Figure 8, we reconstructed the results of the neural network PtyNet-S with different overlap rates as initial guesses after 50 rounds using rPIE and ePIE algorithms. For complex objects, good initial guesses yield better reconstruction results under the conditions of a few iterative rounds and low overlap rates. As shown by the curves in Figure 9, a better initial guess allows the algorithm to converge faster. This indicates that the combination of neural networks and traditional algorithms can greatly improve the efficiency and accuracy of the results. Moreover, the traditional algorithms do not easily fall into local optimal solutions. The fast prediction ability of the neural network and the accuracy of the traditional algrithm combined are ideal to save the experimental time and obtain the imaging information in real time.
