# Supplementary material for: An efficient ptychography reconstruction strategy through fine-tuning of large pre-trained deep learning model
Source: iScience. 2023 Nov 10;26(12):108420. doi: 10.1016/j.isci.2023.108420 (PMC10687283; doi:10.1016/j.isci.2023.108420)
Supplement: Document S1. Figures S1–S9 [file mmc1.pdf]

iScience, Volume *26*

Supplemental information

An efficient ptychography reconstruction strategy through fine-tuning of large pre-trained deep learning model

Xinyu Pan, Shuo Wang, Zhongzheng Zhou, Liang Zhou, Peng Liu, Chun Li, Wenhui Wang, Chenglong Zhang, Yuhui Dong, and Yi Zhang

Supplementary Information

(For “An efficient ptychography reconstruction strategy through fine tuning of large pre-trained deep learning model”)

Super-resolution recovery:

In ptychography experiments, the resolution of the reconstructed image is influenced by various factors, such as the probe size, the acquisition ability of high-frequency signal, and the number of detector pixels. When the wavelength and the wavefront propagation distance are constant, the resolution of the object plane is determined by the number and size of detector pixels. However, using a higher resolution detector to capture the signal would result in a larger computational load for subsequent tasks. To save computational resources, the diffraction map is often center-cropped, while this approach can result in loss of high-frequency signals and imperfect recovered details.

Here, we suggest utilizing super-resolution neural networks to enhance the resolution and improve the accuracy of high-frequency information. Specifically, we employ Enhanced Super-Resolution Generative Adversarial Networks [1](ESRGAN). As depicted in Fig. S3, ESRGAN can significantly enhance image clarity and texture details with an increased image size.

ESRGAN manages to recover high-frequency information and textured structures. In cases where the resolution is compromised or the number of iterations is insufficient, one can quickly locate the ROI and perform finer scanning on the ROI, based on the results obtained from super-resolution network. It should be noted that GAN results are generative and therefore, produce some inaccurate or unfavorable visual effects. Despite the concerns, whether or not the specific structural details recovered by GAN are reliable needs to be eventually determined by the persons conducting the experiments. Therefore, we propose an experimental workflow as shown in Fig. S1. This workflow combines neural networks and traditional algorithms to improve reconstruction efficiency, optimize the visual outcomes of front-end experimenters, and allow for real-time online processing of ptychography object reconstruction. Reliable results requiring subsequent physical analysis will still be reconstructed in the backend, while the neural network predictions as initial guesses will shorten this process and lead to a globally optimal solution. In this paper, we use the ESRGAN model without additional training. Because the simulation data we used are web images and the samples of the experimental data are too small to get a more effective model. In the future, the super-resolution method can be refined after the light source facility generates a large amount of data.

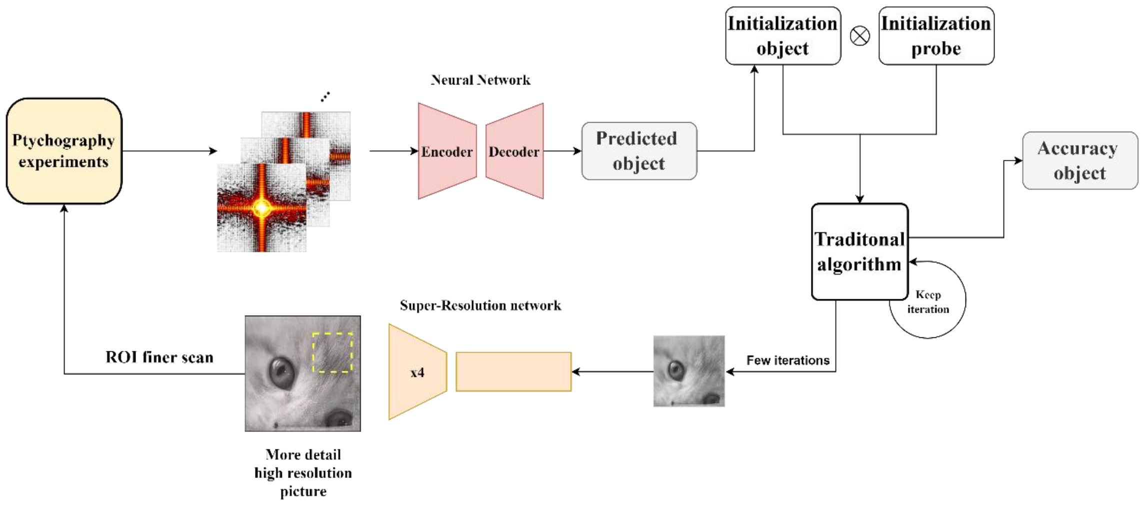

Fig. S1: Reconstruction process of the ptychography experiment combining neural networks and traditional algorithms, related to "Integration with traditional algorithms" in "STAR METHOD" and Figure 8. The combination of neural networks and conventional algorithms gives better resolution reconstruction results.

To validate the feasibility of our approach, we introduced a dataset from the paper[2]. The dataset utilized a Fermat spiral[3] scan with an unknown overlap rate. The scan consisted of a total of 2347 positions, with a diffractogram size of 512×512 and a reconstructed object size of 1606×2355. We selected 2247 scanned positions as the training set for PtyNet-S, and the remaining 100 scanned positions were used as the test dataset. The training method used was the same as main text in results part. The results are depicted in the figure below.

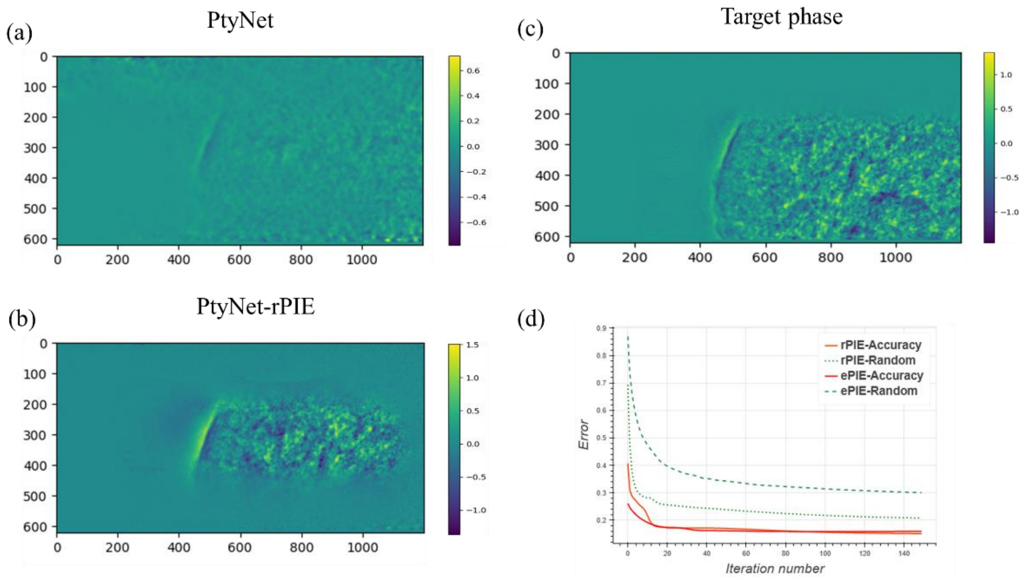

Fig. S2: Partial reconstruction of FCC data , related to Figure 8-9. (a) shows the phase results of the last 100 scanned positions recovered by PtyNet-S. (b) shows the phase results after 20 rounds of iteration using rPIE with the results recovered by PtyNet-S as the initial guess. (c) shows the results of intercepting the last 100 scanned positions after using rPIE to recover the high iteration rounds. (d) shows the error profiles of the network prediction and random initial guesses after rPIE and ePIE iterations. The initial guesses obtained from network prediction can converge faster.

The distribution predicted by the network is used as the initial guess of the conventional algorithm for a few rounds of iteration to obtain images with better clarity. Then, the recovered image is clearer after using the super-resolution network, and the corresponding results are shown in Fig S3. Taking the experimental data as an example, the time of the whole workflow from the proposed network to super-resolution network recovery is about 280 s.

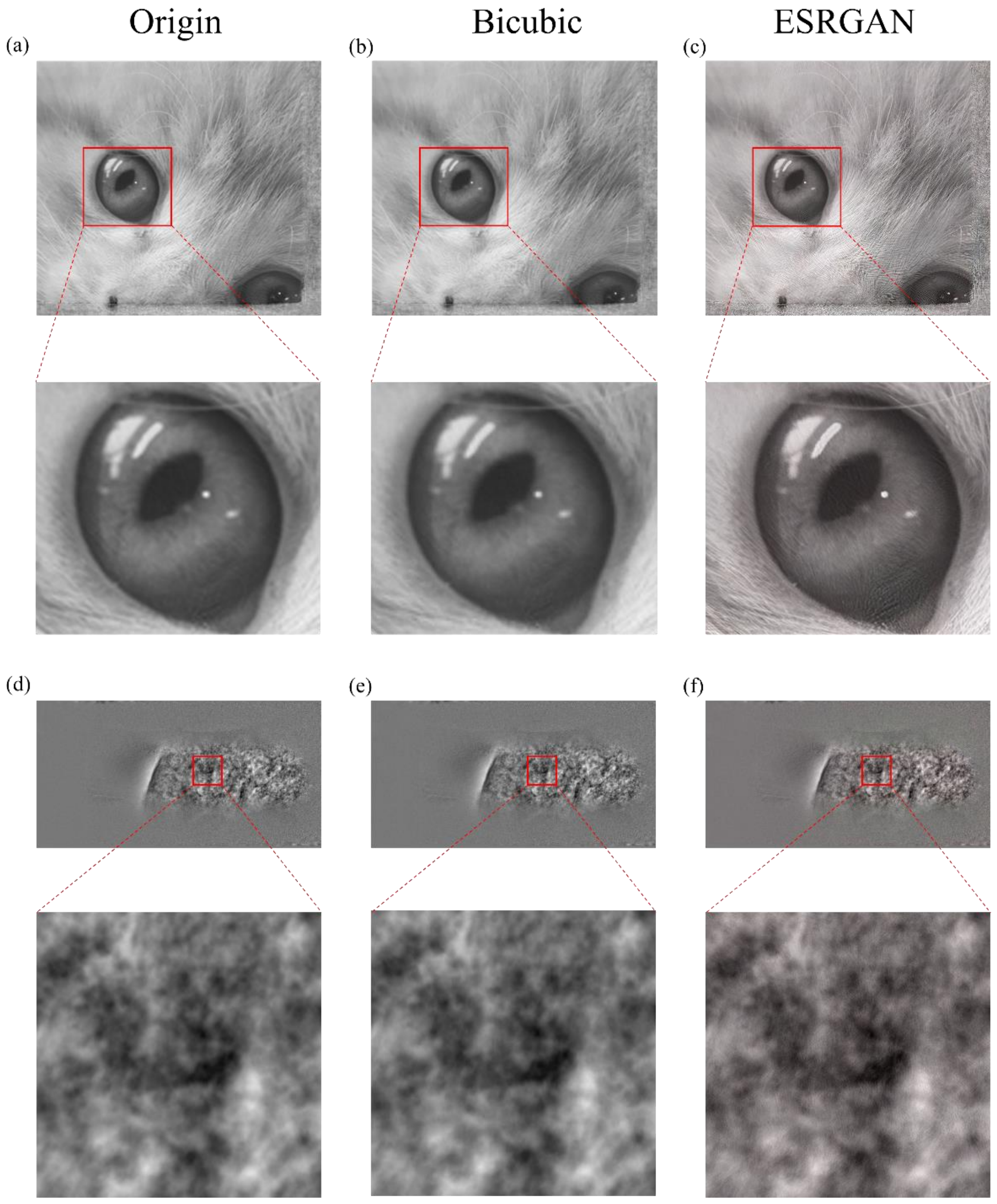

Fig. S3: Super-resolution recovery of reconstruction results, related to Figure 8. (a) and (d) show the phases of the simulated and experimental data after reconstruction using rPIE, and the red boxes are ROI. (b) and (e) show the results after enlarging the reconstructed phases by 4 times using bicubic. (c) and (f) are the results after processing the reconstructed phase using ESRGAN.

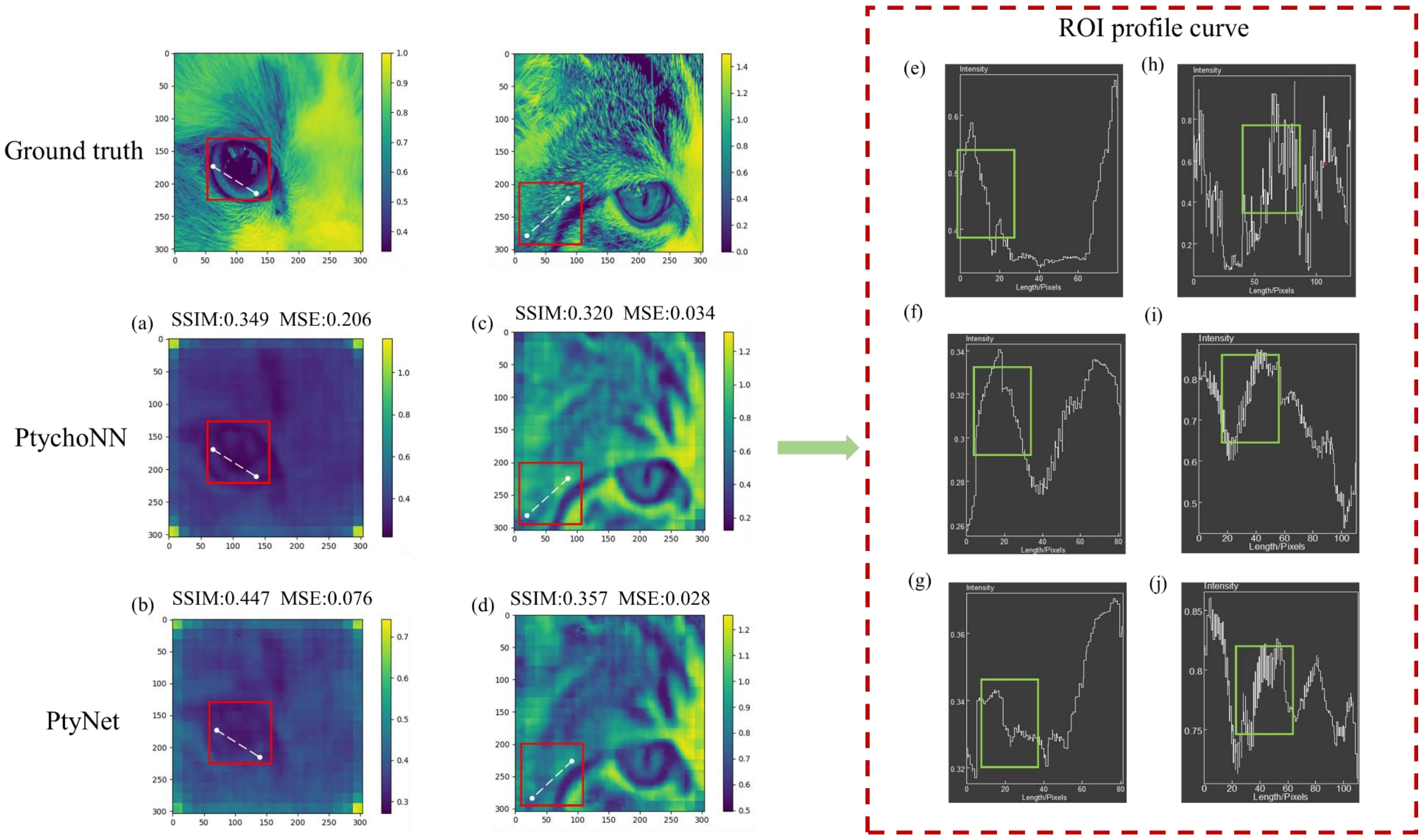

Fig. S4: Comparison of PtyNet-S and PtychoNN in ROI, related to Figure 2. (a) and (c) show the amplitude and phase distributions predicted by PtychoNN, respectively, where the red boxed area is ROI and the white dashed line is Line-profile. (b) and (d) show the amplitude and phase distributions predicted by PtyNet-S, respectively, where the red boxed area is ROI and the white dashed line is Line-profile. (e)-(g) correspond to the Line-profile of (a), (b) and ground truth, respectively, where the PtychoNN has stronger resolution (jump up of pixel values) at the edge of the eye. (h)-(j) correspond to the Line-profile of (c), (d) and ground truth. The PtyNet-S can discern high-frequency (the degree of oscillation of the curve) signals in the hair region.

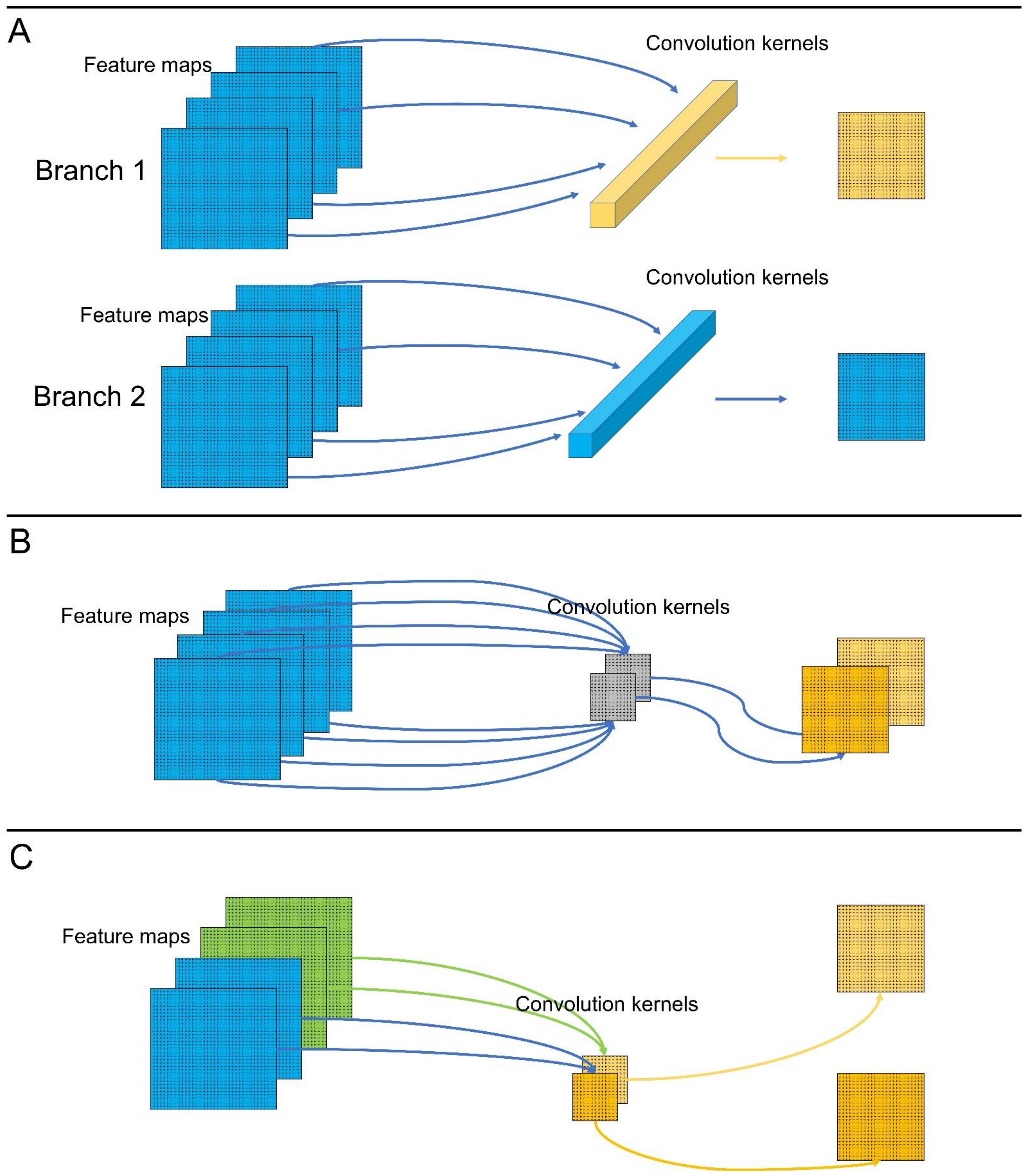

Fig.S5: the schematic architectural design of the three models, related to "Architecture of neural networks" in "METHOD DETAILS". A shows the schematic architecture of PtychoNN. PtychoNN is reconfigured with a two-branch structure in the decoder part, but the number of parameters and the computational resources take up a large amount. B shows the schematic architecture of PtychoNet. PtychoNet is reconfigured with a single-branch structure. C shows the schematic architecture of PtyNet.PtyNet is reconfigured with a single-branch structure. C shows the schematic architecture of PtyNet. PtyNet-S and PtyNet-B adopts a group convolutional architecture in the decoder part, which reduces the computational resource consumption and avoids the interaction between amplitude and phase reconstruction.

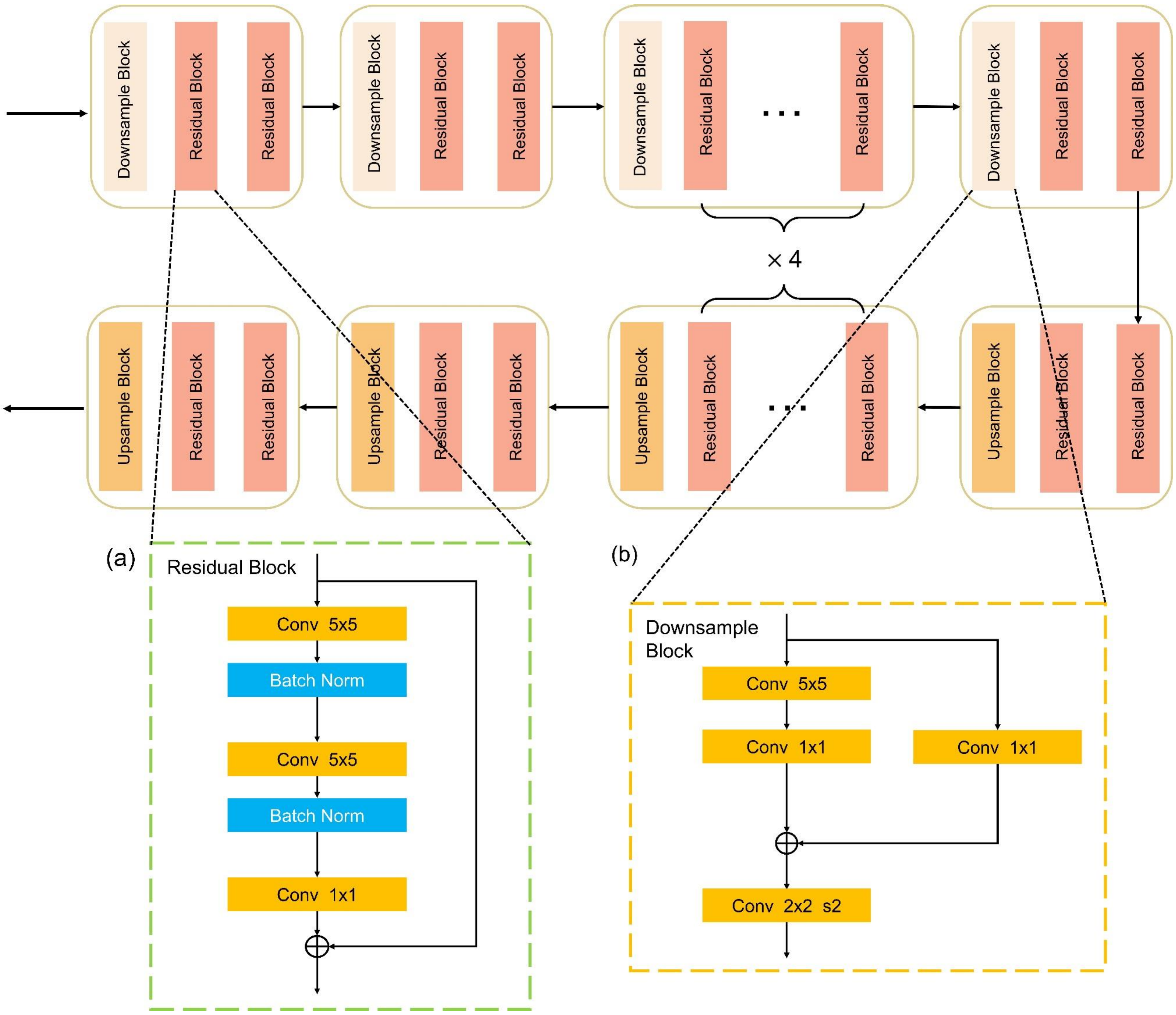

Fig. S6: Architecture diagram of PtyNet-B with about 20 million parameters and schematics of different Blocks, related to "Architecture of neural networks" in "METHOD DETAILS". (a) shows that the Residual Block contains convolution and Batchnorm layers. (b) shows that the Downsample Block contains full convolution, where the step of the downsampling convolution is 2. The Residual Block replaces the convolutional layers in the PtyNet and stack. The Downsample Block replaces the Maxpooling layer. The downsampling convolution is replaced by transposed convolution in the upsampling phase as well as by grouped convolution.

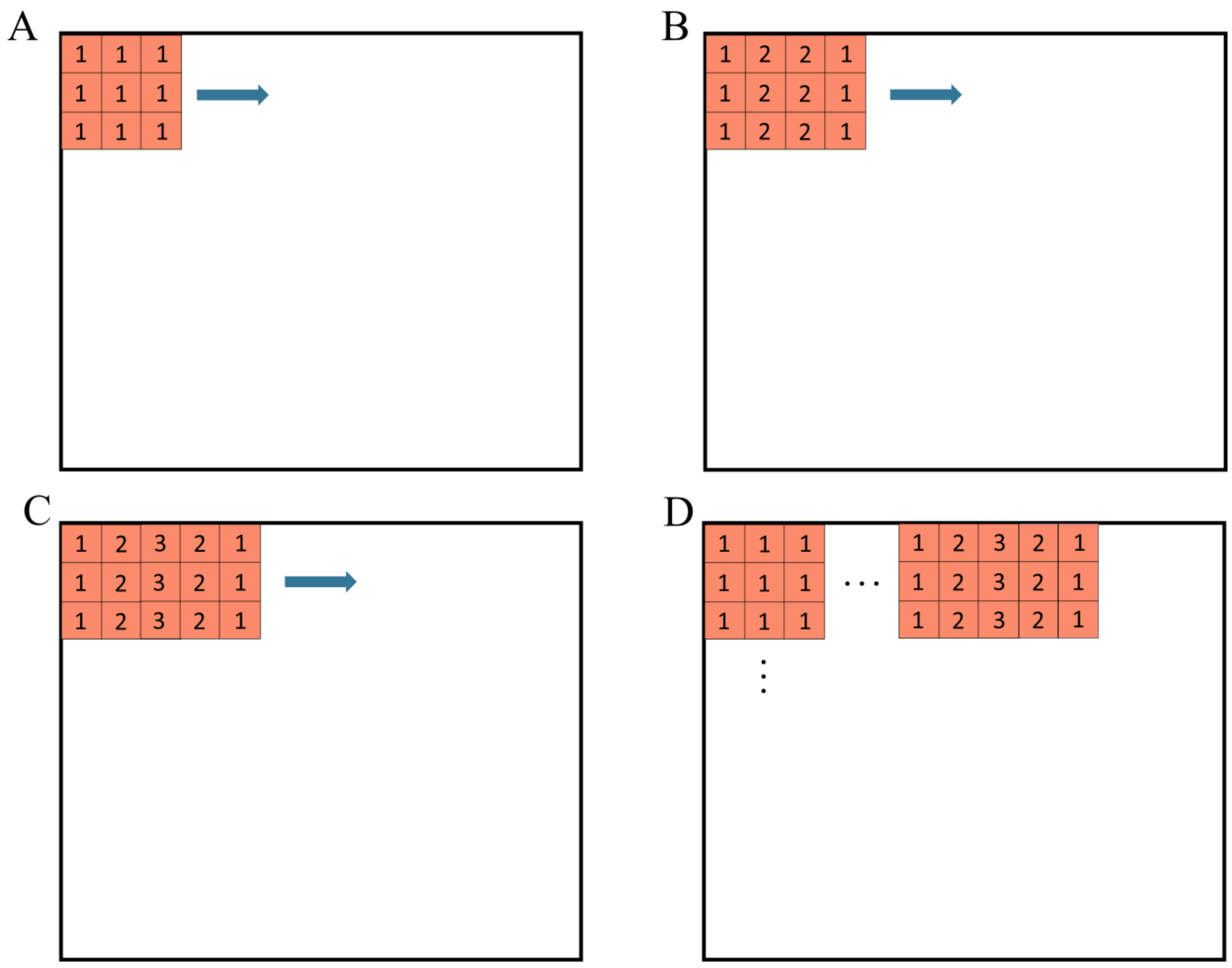

Fig. S7: a schematic diagram of the implementation of the stitching method, related to Figure 1. A full 1 count matrix is used to scan the corresponding position in parallel with the probe, and finally the number of overlaps per pixel is obtained.

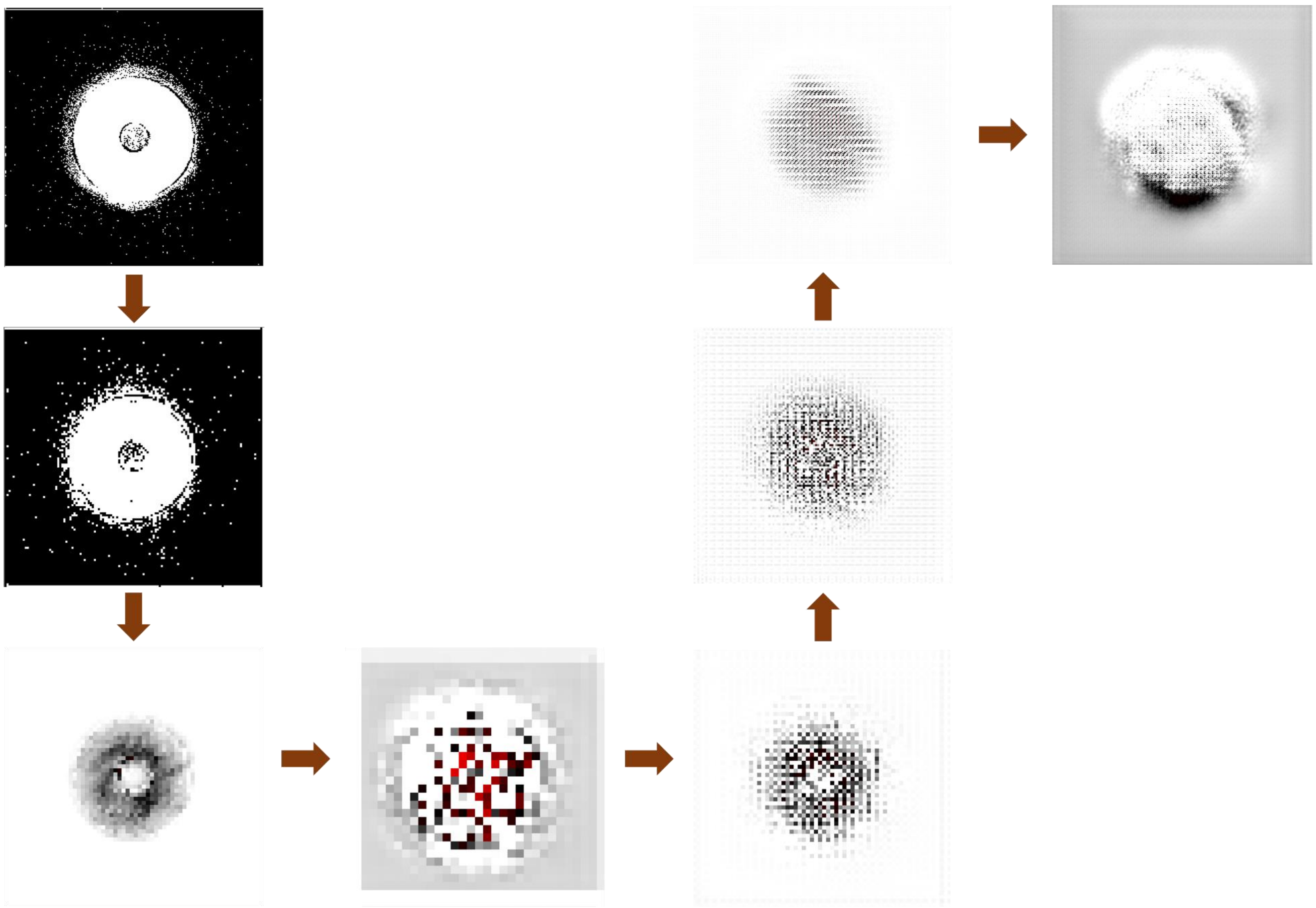

Fig. S8: the diffraction data input to the neural network being used by a convolution operation to extract features and reconstruct the object, related to Figure 3. Where the convolution operation reduces the high frequency information.

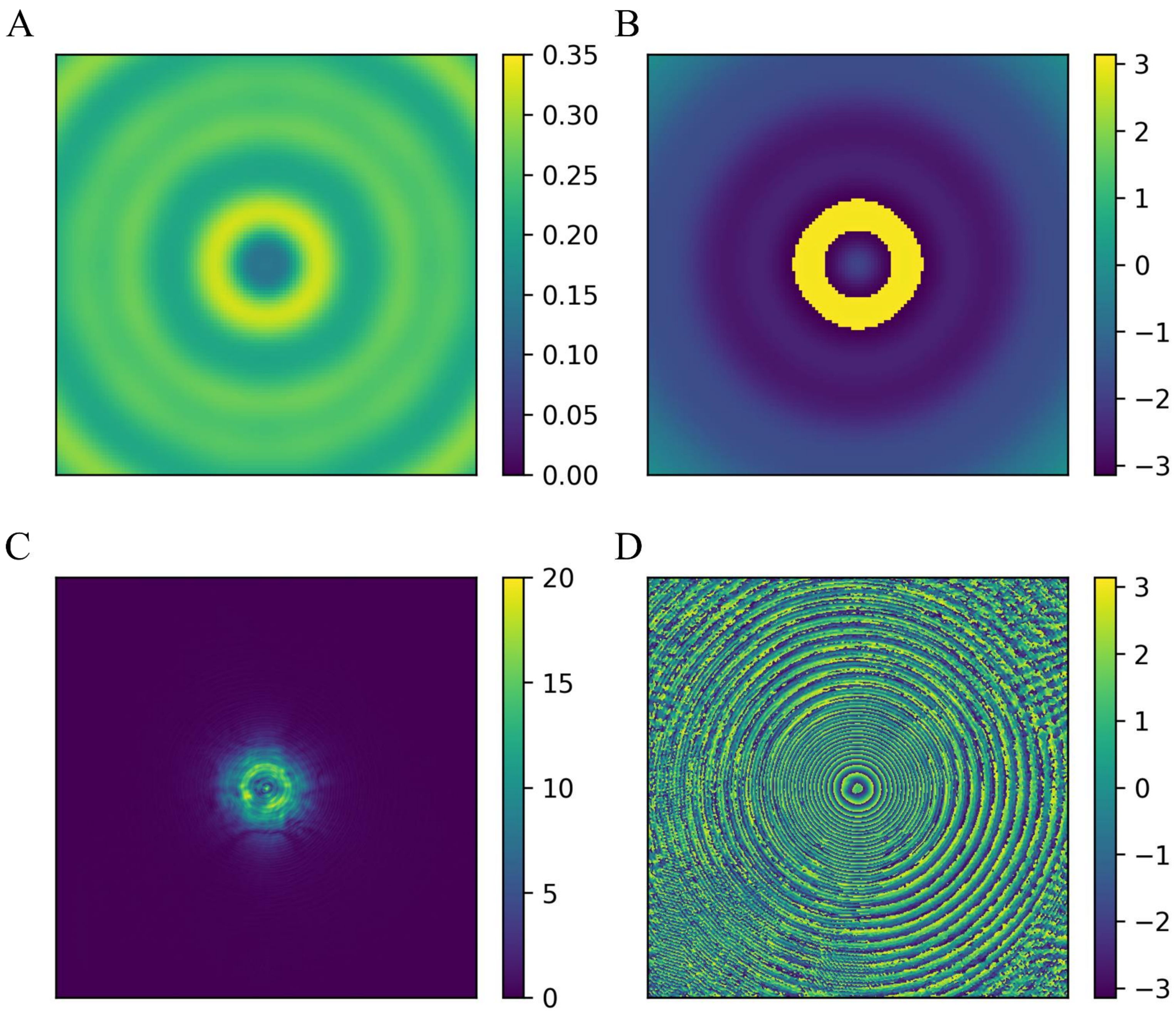

Fig. S9 Simulated probe distribution and FCC's initialized probe distribution., related to "Data simulation" in "METHOD DETAILS" and Figure 5. A and B show the distribution of probe amplitudes and phases in simulated data training PtyNet-S with a size of 128×128. C and D show the initial guess probe distribution for FCC data with a size of 512×512.

References

1. Wang, X., Yu, K., Wu, S., Gu, J., Liu, Y., Dong, C., Qiao, Y., and Change Loy, C. (2018). Esrgan: Enhanced super-resolution generative adversarial networks. In Proceedings of the European conference on computer vision (ECCV) workshops, pp. 0–0.

2. Odstrčil, M., Lebugle, M., Guizar-Sicairos, M., David, C., and Holler, M. (2019). Towards optimized illumination for high-resolution ptychography. Opt. Express *27*, 14981–14997. 10.1364/OE.27.014981.

3. Huang, X., Yan, H., Harder, R., Hwu, Y., Robinson, I.K., and Chu, Y.S. (2014). Optimization of overlap uniformness for ptychography. Opt. Express *22*, 12634–12644. 10.1364/OE.22.012634.
